# Changing Conversations: The Rise of Gender and Sexuality Discourse on Reddit

**DOI:** 10.1007/s10508-024-03051-9

**Published:** 2024-12-27

**Authors:** Philipp Stang, Maren Weiss, Christian Winkler, Stefanie Scholz

**Affiliations:** 1 School of Psychology, SRH University, Fürth, Germany; 2 Institute of Psychology, UMIT Tyrolia, Hall in Tirol, Republic of Austria; 3 Department of Business Administration, University of Technology Nuremberg Georg Simon Ohm, Nuremberg, Germany; 4 School of Applied Sciences for Health, Education and Social Sciences, SRH University, Merkurstr 19, 90763 Fürth, Germany

Scientific disciplines, such as psychology and sexology, now understand sexuality and gender(-ness) as a spectrum with multiple manifestations across the lifespan (Coleman et al., 2022; Dawson-Squibb et al., 2023; Nieder & Strauß, 2018, 2021; Voß, 2021, 2023; World Medical Association [WMA], 2017). Sociopsychologically, it appears that changes are also occurring in the representation of sexuality and gender in the context of new media. However, the current research situation does not reflect these new perspectives on sexuality and gender.

## Theoretical and Empirical Foundations

Social change has an impact on psychosexual development and presents people with new developmental challenges. The paradigm shift in gender as well as the media presence of different genders also gives the impression that gender and gender diversity are constructs that are highly relevant for (young) people (Lesben- und Schwulenverband [LSVD] e.V.). Gender and sexual diversity are more common in younger generations (Gallup, 2023). Queer and trans are reported at 19.7% for Generation Z (Gallup, 2023); 5% of young adults in the USA say that their gender is different from their assigned gender at birth (Brown, 2022). Non-heteronormative identities are estimated at 7.2% for the USA in 2022; 2012: < 5% (Gallup, 2023). In 2021, more people, 26%, knew a non-binary person than in 2018, 18% (Brown, 2022). The new ICD-11 diagnostic manual reflects these changes by deleting some gender-related disorders and, in general, a more liberal approach to sexual behaviors.

In addition to developmental psychological challenges, i.e., regarding the development of gender identity, sexuality(ies), gender(s), and relationship(s) are also linked to mental and physical health, life satisfaction, and quality of life (Buddeberg, 2005; Fahrenberg et al., 2000; Freund & Riedel-Breidenstein, 2020; Stang & Ondrejtschak, in press; World Health Organization [WHO], 2002).

For people, communicating sexuality- and gender-related aspects are ways of entering into interpersonal dialogue. In doing so, individuals also use the psychological functions of decatastrophization, validation, and normalization by other communication partners in relation to sexual- and gender-related aspects of their own identity. New media, such as chat platforms, provide an opportunity to get in touch with other people with many economic advantages while remaining anonymous at the same time. Reddit is a social news aggregator that offers the possibility of interpersonal exchange.

In addition, Reddit is one of the fastest growing social platforms and plays a significant role in the communication and discussion of topics, especially among Generation Z (see supplement Fig. 1). This platform offers a variety of subreddits that focus on specific topics and allows users to create, share, and discuss content. Reddit’s structure encourages in-depth discussions and sharing of information, making it a valuable resource for exploring topics such as sexuality and gender. The platform also allows users to discuss in a relatively anonymous environment, which is particularly important when it comes to sensitive topics such as sexuality and gender. This makes Reddit a valuable source of data for researchers who want to study the attitudes, experiences, and discussions surrounding these topics.

**Fig. 1:**
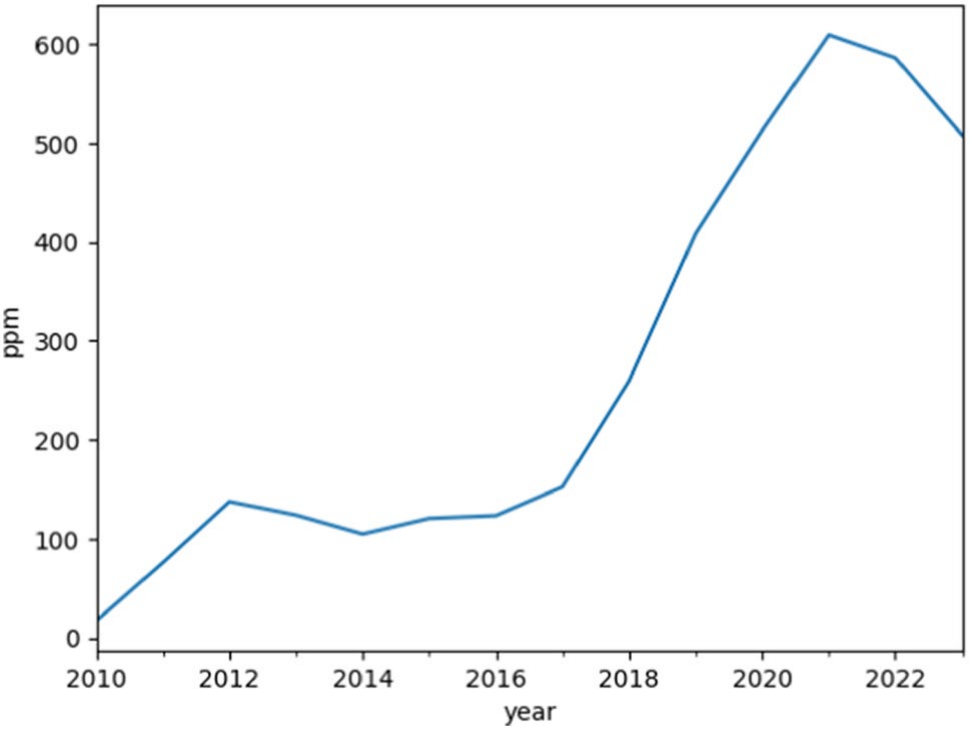
Posts of the aggregated, relevant subreddit per one million Reddit posts (parts per million)

### State of Research

The current state of research shows studies that have dealt with the specific social media platform Reddit and clinical-psychological, social-psychological, and also differential/personality-psychological aspects (Abavi et al., 2020; Association for Computational Linguistics, 2021; Bak et al., 2023; Blackburn et al., 2018; Cavazos-Rehg et al., 2019; Gkotsis et al., 2020; Himelein-Wachowiak et al., 2022; Huguet-Cabot et al., 2021; Jonason & Tome, 2019; Komarnicky et al., 2019; Lavis & Winter, 2020; Park & Conway, 2017; Ramseyer Winter et al., 2018; Sowles et al., 2018; Strand & Gustafsson, 2020; Zemla et al., 2017; Zimmer & Imhoff, 2020). Studies in the context of sexuality and/or gender in the context of Reddit dealt with a variety of topics such as experiences of sexually assaultive behavior (Abavi et al., 2020; Brennan et al., 2018), masturbation abstinence (Zimmer & Imhoff, 2020), contraceptive use and sexual health (Ramseyer Winter et al., 2018), and genital self-image and body-related concerns (Kormanicky et al., 2019). Reddit research also covers relationship issues such as the role of sexuality for relationship happiness (Jonason & Tome, 2019) and relationship help-seeking by men and women (Entwistle et al., 2021). Reddit is also cited by adult Australian trans people as the most popular online resource for health information (Bretherton et al., 2021).

The current state of research shows that sexuality and gender are topics that users of the Reddit platform discuss with varying degrees of focus. However, whether this is a recent phenomenon and whether there has been a change in the frequency of communicative exchange over time cannot be deduced from the current state of research.

### Research Question

Our study examined to what extent users use a platform such as Reddit to exchange views on sexuality- and gender-related aspects, and whether changes can be observed over the years.

## Method

### Measures and Procedure

The Pushshift.io tool (https://pushshift.io/) was used to acquire the content of relevant subreddits for the analysis. Pushshift is a data archiving service that provides an extensive collection of historical Reddit data, including posts, comments, and metadata. The data were downloaded and extracted for the entire period of the respective subreddits (these were created at different times) up to and including May 31, 2023. This period was selected to capture current trends and discussions while ensuring a sufficient amount of data for robust analysis.

For this study, the focus of the data analysis was deliberately placed on the top-level posts, i.e., the original posts in the selected subreddits. Comments following these posts were not included in the analysis. This methodological decision was made for several reasons:

1. Top-level posts represent the initial topics and questions raised by users and around which the discussions are structured. These posts provide a direct and clear representation of users’ concerns, opinions, and experiences without being diluted by the often complex and divergent discussions in the comments.
2. Including comments would have significantly increased the amount of data and made the analysis much more complex. Comments often contain nested discussions that mix different topics and perspectives in one thread.
3. By focusing on top-level posts, a certain homogeneity in the analyzed data was ensured, as all analyzed content had the same structural starting point. This enabled a comparable analysis of the topics and language patterns across the various subreddits.

The acquisition of the data from the respective start of the subreddits up to and including May 31, 2023, comprised the following steps:

1. Based on the research topic, eight subreddits were selected that dealt intensively with issues of sexuality and gender. The following subreddits were selected (in alphabetical order): r/bisexual, r/NonBinary, r/NonBinaryTalk, r/Puberty, r/queer, r/sexeducation, r/trans, r/transtimelines. The selection of subreddits was based on their relevance to the research topics of sexuality and gender as well as their active user base. The selected subreddits covered a variety of perspectives and experiences that were of interest for analyzing the social construction of gender and sexuality. The large number of posts and different authors ensured a broad database for statistical analyses and made it possible to identify general trends and specific discourse structures.
2. Using Pushshift, an API query was created for each of the subreddits mentioned to download the posts and their associated metadata, including post title, content, author, and timestamp. The API requests were configured to cover the entire defined time period and extract all available data within that time frame.
3. After the download, the data were cleansed to remove duplicate entries and ensure that only relevant posts were included in the analysis. Due to the fact that each subreddit had its own moderation, the content was already curated or cleansed so that spam was either identified and removed by Reddit’s own algorithms or deleted by the respective moderation. The data were also saved in a structured format in order to prepare it for the subsequent quantitative analysis.

## Results

### Evaluation of the Development of the Posting Volume

The subreddit “r/sexeducation” had a rather low total volume of 6097 top-level posts by 2441 authors in the range of topics covered here. There was a continuous increase from 2019 to over 200 posts per month until 2023. The number of authors had also been rising continuously since 2018, with fluctuations. The subreddit “r/Puberty” had 37,582 initial posts (authors: 7375). After a sharp increase in top-level posts and the number of authors from 2019 to 2020, the trend stagnated at a fluctuating but constant average level of around 800 initial posts per month.

The “r/trans” subreddit contained 326,372 initial posts by 84,227 authors. A significant increase in posts can be observed in the period from 2019 to 2023, from under 2000 posts per month to over 12,000; peak: 2022 with a brief dip in autumn 2021 (see supplement Fig. 2).

The posting volume of the “r/transtimelines” subreddit totaling 75,301 (authors: 14,225) was smaller but can be understood as more topic-specific. In this subreddit, transition processes are documented and commented on over time by those affected. There has been a constant increase in top-level posting behavior since the beginning, which has increased significantly since 2017 and continued until the end of the analysis period at the end of May 2023 with up to around 1,500 posts per month. The number of authors also increased accordingly over this period.

The posting volume of the “r/NonBinaryTalk” subreddit appears to be rather lower, totaling 15,507 (authors: 5858), with a peak in 2021 to mid-2022 at around 500 posts per month. In contrast, the “r/NonBinary” subreddit has a total posting volume of 159,701 (authors: 40,276), with the peak in both the number of monthly top-level posts (around 4500) and authors being reached in 2020/2021 and decreasing significantly since then.

The “r/queer” subreddit had a total posting volume of 7697 from a total of 3124 authors. Overall, it is clear that although this subreddit is relatively small, it has been growing in importance since 2020.

The “r/bisexual” subreddit had a total posting volume of 249,366 top-level posts (authors: 73,352). After a steep increase in initial monthly posts from 2019 to 2020 to almost 6000 posts per month, the posting volume has declined significantly since then, as has the development of the number of authors.

### Relative Growth of the Subreddits in Relation to the Total Growth of All Top-Level Posts on Reddit

After looking at the development of the absolute top-level and author numbers, it is worth taking a look at the development of posting activity in the selected subreddits in relation to the top-level posts on the entire Reddit platform.

The scale was chosen to show the growth of the relevant subreddits in “parts per million” (ppm), i.e., how many posts in the respective (or later aggregated), relevant subreddits account for one million top-level posts on Reddit as a whole. This presentation makes it possible to quantify and compare the growth of activity in the relevant subreddits in relation to overall activity on Reddit.

Looking at the relative growth of the individual subreddits, it is noticeable that almost all of them have grown disproportionately fast in 2021 compared to the overall growth of top-level posts on Reddit. While Reddit as a whole grew, the relative number of posts in these subreddits increased even more since 2017, indicating an increasing focus and activity of users on the topics discussed (see Fig. 1). Growth peaked in 2020 and 2021 in particular, which could be due to increased activity and possibly also global events such as the COVID-19 pandemic, which intensified online discussions in these areas. The curve shows a slight decline in 2022. This could be due to various factors, such as lower online activity after the COVID-19 restrictions were lifted, including on www.youtube.com (Statista, 2024). It is also possible that other platforms have gained in importance, leading to a decline in activity on these subreddits.

## Discussion

The extensive posting volume of subreddits in the context of transgender and non-binary genders can be explained in the context of the gender paradigm shift (American Psychological Association [APA], 2015; Coleman et al., 2022; Dawson-Squibb et al., 2023; Nieder & Strauß, 2018, 2021; Voß, 2021, 2023; World Medical Association [WMA], 2017). It can be assumed that younger users are more likely to use Reddit and that there is an increase in gender diversity in the younger generations compared to older generations (Brown, 2022; Gallup, 2023). Our data confirm that Reddit is an important online resource for transgender and non-binary genders, especially for Generation Z (Bretherton et al., 2021; Krebs, 2022). Considering the number of people who follow the corresponding postings, it can be assumed that this platform has a wide reach in the area of gender and sexual topics. With reference to the current state of research, communicative exchange via Reddit also appears to have advantages for specific groups of people compared to traditional counseling and exchange opportunities (Entwistle et al., 2021; Xu et al., 2023). Already Bretherton et al. (2021) referred to the preference of trans people to share health information via online resources.

High posting volumes were found for subreddits in the context of gender diversity, particularly for specific topics. In contrast, rather low posting volumes were found for specific sexual preferences. An increase in posting volumes can be observed, particularly around 2019–2020, with the increase in postings to the “bisexual” subreddit taking place somewhat earlier than to the “non-binary” and “trans” subreddits.

Social media offer both risks and benefits for human psychological functioning. Known risks include non-substance-related addiction, social withdrawal from the “offline” world, increased social comparison, and subculture orientation. However, with regard to sexual- and gender-related topics, the possibility of anonymous communication can be seen as a positive aspect. In addition, people can get in touch with others who belong to a sexual or gender diversity without having to out themselves by name, even if they are physically far away.

Social media, such as Reddit, also offer new data collection methods for research (e.g., via big data analyses), and the opportunity to discuss specific research interests. The potential of Reddit analyses and comparable approaches is certainly far from exhausted.

## Supplementary Information

Below is the link to the electronic supplementary material.

Supplementary file1 (DOCX 118 KB)

## Data Availability

Data are available in that repository: https://github.com/data-for-health

## Code Availability

Code is available upon request.

## References

[CR1] Abavi, R., Branston, A., Mason, R., & Du Mont, J. (2020). An exploration of sexual assault survivors’ discourse online on help-seeking. *Violence and Victims,**35*(1), 126–140. 10.1891/0886-6708.VV-D-18-00148 32015073 10.1891/0886-6708.VV-D-18-00148

[CR2] American Psychological Association. (2015). Guidelines for psychological practice with transgender and gender nonconforming people. *American Psychologist,**70*(9), 832–864. 10.1037/a0039906 26653312 10.1037/a0039906

[CR3] Association for Computational Linguistics. (Ed.) (2021). *Proceedings of the 16th conference of the European Chapter of the Association for Computational Linguistics: Main Volume*. arXiv. 10.48550/arXiv.2101.11956

[CR4] Bak, M., Chiu, C., & Chin, J. (2023). Mental health pandemic during the COVID-19 outbreak: Social media as a window to public mental health. *Cyberpsychology, Behavior and Social Networking,**26*(5), 346–356. 10.1089/cyber.2022.0116 37057976 10.1089/cyber.2022.0116

[CR5] Blackburn, K. G., Yilmaz, G., & Boyd, R. L. (2018). Food for thought: Exploring how people think and talk about food online. *Appetite,**123*, 390–401. 10.1016/j.appet.2018.01.022 29407531 10.1016/j.appet.2018.01.022

[CR33] Brennan, C. L., Swartout, K. M., Cook, S. L., & Parrott, D. J. (2018). A qualitative analysis of offenders’ emotional responses to perpetrating sexual assault. *Sexual Abuse: A Journal of Research and Treatment*, *30*(4), 393–412. 10.1177/1079063216667917 10.1177/1079063216667917 27591752

[CR6] Bretherton, I., Thrower, E., Zwickl, S., Wong, A., Chetcuti, D., Grossmann, M., Zajac, J. D., & Cheung, A. S. (2021). The health and well-being of transgender Australians: A national community survey. *LGBT Health,**8*(1), 42–49. 10.1089/lgbt.2020.0178 33297824 10.1089/lgbt.2020.0178 PMC7826417

[CR7] Brown, A. (2022). *About 5% of young adults in the U.S. say their gender is different from their sex assigned at birth*. https://www.pewresearch.org/short-reads/2022/06/07/about-5-of-young-adults-in-the-u-s-say-their-gender-is-different-from-their-sex-assigned-at-birth/

[CR34] Buddeberg, C. (2005). *Sexualberatung*. Georg Thieme Verlag. 10.1055/b-002-21516

[CR8] Cavazos-Rehg, P., Grucza, R., Krauss, M. J., Smarsh, A., Anako, N., Kasson, E., Kaiser, N., Sansone, S., Winograd, R., & Bierut, L. J. (2019). Utilizing social media to explore overdose and HIV/HCV risk behaviors among current opioid misusers. *Drug and Alcohol Dependence,**205*, 107690. 10.1016/j.drugalcdep.2019.107690 31778902 10.1016/j.drugalcdep.2019.107690 PMC6894427

[CR9] Coleman, E., Radix, A. E., Bouman, W. P., Brown, G. R., de Vries, A. L. C., Deutsch, M. B., Ettner, R., Fraser, L., Goodman, M., Green, J., Hancock, A. B., Johnson, T. W., Karasic, D. H., Knudson, G. A., Leibowitz, S. F., Meyer-Bahlburg, H. F. L., Monstrey, S. J., Motmans, J., Nahata, L., … Arcelus, J. (2022). Standards of care for the health of transgender and gender diverse people, version 8. *International Journal of Transgender Health,**23*(Suppl 1), S1–S259. 10.1080/26895269.2022.2100644 36238954 10.1080/26895269.2022.2100644 PMC9553112

[CR10] Dawson-Squibb, J.-J., Davids, E. L., Viljoen, M., Rice, K., & Stein, D. J. (2023). The WHO international classification of diseases 11th revision (ICD-11). In J. L. Matson (Ed.), *Handbook of clinical child psychology: Integrating theory and research into practice* (pp. 53–78). Springer International Publishing. 10.1007/978-3-031-24926-6_4

[CR11] Entwistle, C., Horn, A. B., Meier, T., & Boyd, R. L. (2021). Dirty laundry: The nature and substance of seeking relationship help from strangers online. *Journal of Social and Personal Relationships,**38*(12), 3472–3496. 10.1177/02654075211046635 34924670 10.1177/02654075211046635 PMC8669208

[CR35] Fahrenberg, J., Myrtek, M., Schumacher, J., & Brähler, E. (2000). *Fragebogen zur Lebenszufriedenheit (FLZ): Handanweisung*. Hogrefe.

[CR36] Freund, U., & Riedel-Breidenstein, D. (2020). *Sexuelle Übergriffe unter Kindern: Handbuch zur Prävention und Intervention*. Mebes & Noack.

[CR12] Gallup. (2023). *U.S. LGBT Identification Steady at 7.2%*. https://news.gallup.com/poll/470708/lgbt-identification-steady.aspx

[CR13] Gkotsis, G., Mueller, C., Dobson, R. J. B., Hubbard, T. J. P., & Dutta, R. (2020). Mining social media data to study the consequences of dementia diagnosis on caregivers and relatives. *Dementia and Geriatric Cognitive Disorders,**49*(3), 295–302. 10.1159/000509123 32854092 10.1159/000509123

[CR14] Himelein-Wachowiak, M., Giorgi, S., Kwarteng, A., Schriefer, D., Smitterberg, C., Yadeta, K., Bragard, E., Devoto, A., Ungar, L., & Curtis, B. (2022). Getting “clean” from nonsuicidal self-injury: Experiences of addiction on the subreddit r/selfharm. *Journal of Behavioral Addictions,**11*(1), 128–139. 10.1556/2006.2022.00005 35312631 10.1556/2006.2022.00005 PMC9109623

[CR15] Huguet-Cabot, P.-L., Abadi, D., Fischer, A., & Shutova, E. (2021). Us vs. them: A dataset of populist attitudes, news bias and emotions. In Association for Computational Linguistics (Ed.), *Proceedings of the 16th conference of the European Chapter of the Association for Computational Linguistics: Main Volume* (pp. 1921–1945). arXiv.

[CR16] Jonason, P. K., & Tome, J. (2019). How happiness expectations relate to the Dark Triad traits. *Journal of Social Psychology,**159*(4), 371–382. 10.1080/00224545.2018.1529652 30307810 10.1080/00224545.2018.1529652

[CR17] Komarnicky, T., Skakoon-Sparling, S., Milhausen, R. R., & Breuer, R. (2019). Genital self-image: Associations with other domains of body image and sexual response. *Journal of Sex & Marital Therapy,**45*(6), 524–537. 10.1080/0092623X.2019.1586018 30836857 10.1080/0092623X.2019.1586018

[CR18] Krebs, A. (2022). *The 90–9–1 principle is fascinating* [Tweet]. Twitter. https://twitter.com/krebs_adrian/status/1481335539685072904?s=20

[CR19] Lavis, A., & Winter, R. (2020). #online harms or benefits? An ethnographic analysis of the positives and negatives of peer-support around self-harm on social media. *Journal of Child Psychology and Psychiatry,**61*(8), 842–854. 10.1111/jcpp.13245 32459004 10.1111/jcpp.13245

[CR20] Nieder, T. O., & Strauß, B. (Eds.) (2018). *Geschlechtsinkongruenz, Geschlechtsdysphorie und Trans-Gesundheit: S3-Leitlinie zur Diagnostik, Beratung und Behandlung*. https://register.awmf.org/assets/guidelines/138-001l_S3_Geschlechtsdysphorie-Diagnostik-Beratung-Behandlung_2019-02.pdf

[CR21] Nieder, T. O., & Strauß, B. (2021). *Geschlechtsinkongruenz, geschlechtsdysphorie und trans-gesundheit*. Nomos Verlagsgesellschaft mbH & Co. KG. 10.30820/9783837977585

[CR22] Park, A., & Conway, M. (2017). Longitudinal changes in psychological states in online health community members: Understanding the long-term effects of participating in an online depression community. *Journal of Medical Internet Research,**19*(3), e71. 10.2196/jmir.6826 28320692 10.2196/jmir.6826 PMC5379019

[CR23] Ramseyer Winter, V., Ruhr, L., Pevehouse, D., & Pilgrim, S. (2018). Exploring body image, contraceptive use, and sexual health outcomes among an ethnically diverse sample of women. *Archives of Sexual Behavior,**47*(3), 715–723. 10.1007/s10508-017-1121-3 29305774 10.1007/s10508-017-1121-3

[CR24] Sowles, S. J., McLeary, M., Optican, A., Cahn, E., Krauss, M. J., Fitzsimmons-Craft, E. E., Wilfley, D. E., & Cavazos-Rehg, P. A. (2018). A content analysis of an online pro-eating disorder community on Reddit. *Body Image,**24*, 137–144. 10.1016/j.bodyim.2018.01.001 29414146 10.1016/j.bodyim.2018.01.001 PMC5869127

[CR37] Stang, P., & Ondrejtschak, C. (in press). Sexualtherapie im Kindes- und Jugendalter. In U. Özdemir & J. Velten (Eds.), *Kognitiv-verhaltenstherapeutische Sexualtherapie*. Hogrefe.

[CR25] Statista. (2024). *Anzahl der Visits von youtube.com von Mai 2019 bis Juli 2024 (in Milliarden)*. https://de.statista.com/statistik/daten/studie/1021459/umfrage/anzahl-der-visits-pro-monat-von-youtube/

[CR26] Strand, M., & Gustafsson, S. A. (2020). Mukbang and disordered eating: A netnographic analysis of online eating broadcasts. *Culture, Medicine and Psychiatry,**44*(4), 586–609. 10.1007/s11013-020-09674-6 32277331 10.1007/s11013-020-09674-6 PMC7497418

[CR27] Voß, H.-J. (2021). *The intricacy of the human sexes*. Psychosozial-Verlag. 10.30820/9783837978063

[CR28] Voß, H.-J. (2023). *Einführung in die sexualpädagogik und sexuelle bildung: Basisbuch für studium und weiterbildung* (1st ed.). Urban-Taschenbücher. Verlag W.

[CR40] World Health Organization. (2002). *Defining sexual health: Report of a technical consultation on sexual health*. https://www.cesas.lu/perch/resources/whodefiningsexualhealth.pdf

[CR29] World Medical Association. (2017). *WMA Statement on Transgender People*. https://www.wma.net/policies-post/wma-statement-on-transgender-people/

[CR30] Xu, Q., McMann, T., Godinez, H., Nali, M. C., Li, J., Cai, M., Merenda, C., Lee, C., Araojo, R., & Mackey, T. K. (2023). Impact of COVID-19 on HIV prevention access: A multi-platform social media infodemiology study. *AIDS and Behavior,**27*(6), 1886–1896. 10.1007/s10461-022-03922-z 36471205 10.1007/s10461-022-03922-z PMC9734820

[CR31] Zemla, J. C., Sloman, S., Bechlivanidis, C., & Lagnado, D. A. (2017). Evaluating everyday explanations. *Psychonomic Bulletin & Review,**24*(5), 1488–1500. 10.3758/s13423-017-1258-z 28275989 10.3758/s13423-017-1258-z

[CR32] Zimmer, F., & Imhoff, R. (2020). Abstinence from masturbation and hypersexuality. *Archives of Sexual Behavior,**49*(4), 1333–1343. 10.1007/s10508-019-01623-8 32130561 10.1007/s10508-019-01623-8 PMC7145784

