# Supplementary material for: Changing Conversations: The Rise of Gender and Sexuality Discourse on Reddit
Source: Arch Sex Behav. 2024 Dec 27;54(1):1–5. doi: 10.1007/s10508-024-03051-9 (PMC11782437; doi:10.1007/s10508-024-03051-9)
Supplement: Supplementary file 1 — Supplementary file1 (DOCX 118 KB) [file 10508_2024_3051_MOESM1_ESM.docx]

Supplement

Supplement Table 1: Table 1: Selected subreddits and their description and number of members (as of August 2024)

Supplement Figure 1: Reddit as one of the most trusted sources for Generation Z (source: https://web.swipeinsight.app/posts/how-search-marketers-can-keep-up-with-gen-z-reddit-report-7746)

Supplement Figure 2: Subreddit trans, 2013-2023 (own illustration)

Supplement materials

Supplement Tables:

Supplement Table 2: Selected subreddits and their description and number of members (as of August 2024)

| Subreddit | Description | Members (by 15 August 2024) |
| --- | --- | --- |
| r/bisexual | This group is for discussion and support for those who fall in between, for the "shades of gay" in what is often assumed to be one or the other: * bisexuals * pansexuals * omnisexuals * queers * non-straight individuals ... or anyone who doesn't quite fit the otherwise binary "straight" and "gay" pattern. If you can't work out if you're straight, gay, or anywhere in between... you should probably visit us. | 592.730 |
| r/NonBinary | a culture of varied awesomeness.  A subreddit for people of every stripe who feel that they don't fit into a preference-binary or gender-binary culture. | 251.555 |
| r/NonBinaryTalk | Welcome! This subreddit is a 'general forum' where non-binary people can come together to socialise casually and discuss any topic. Politics are prohibited. Talking about politics is discouraged and should be directed to /r/genderqueer instead. | 37.687 |
| r/Puberty | Community to discuss everything related to puberty. --DMing is strictly forbidden-- | 22.586 |
| r/queer | We're here, we're Queer, get used to it! An open forum to discuss and share things of interest to the Queer/LGBTQIA+ Community | 33.659 |
| r/sexeducation | For discussion of education for sex and relationships. This includes teaching and learning about emotional, social, and physical aspects of having relationships, engaging in sex, sexuality and sexual health across the lifespan. | 20.876 |
| r/trans | Welcome to r/trans! This is a safe space for transgender people to discuss their lives and issues that surround them, find affirmation, and discover community. This is a radically inclusive subreddit for everyone under the trans umbrella; if you are binary, non-binary, genderqueer, agender, GNC, questioning, or any combination, this space is for you. You don't need to be trans to participate here, but this is not the place for questions or statements of opinion from outside of the community. | 538.340 |
| r/transtimelines | A subreddit for sharing your transition timelines. | 236.185 |

Supplement Figures


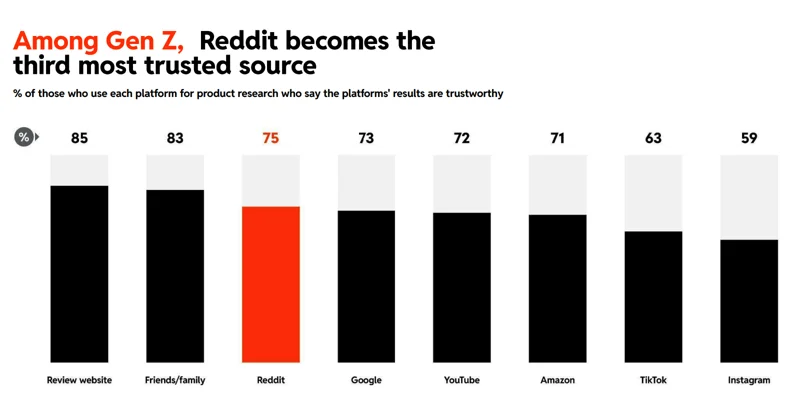


Supplement Figure 1: Reddit as one of the most trusted sources for Generation Z (source: https://web.swipeinsight.app/posts/how-search-marketers-can-keep-up-with-gen-z-reddit-report-7746)


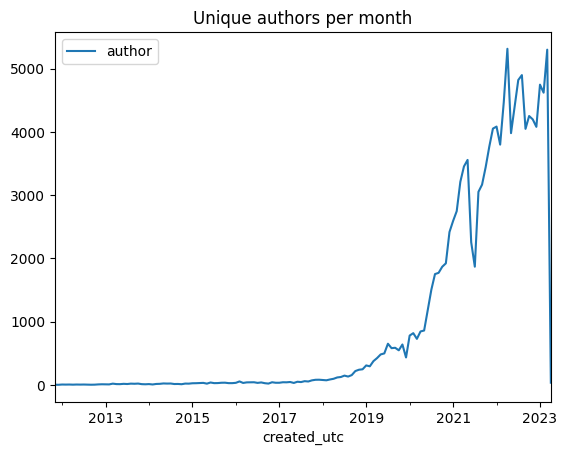


Supplement Figure 2: Subreddit trans, 2013-2023 (own illustration)
